# Social Reward Questionnaire—Adolescent Version and its association with callous–unemotional traits

**DOI:** 10.1098/rsos.160991

**Published:** 2017-04-19

**Authors:** Lucy Foulkes, Craig S. Neumann, Ruth Roberts, Eamon McCrory, Essi Viding

**Affiliations:** 1 Developmental Risk and Resilience Unit, Division of Psychology and Language Sciences, University College London, 26 Bedford Way, London WC1H 0AP, UK; 2 Department of Psychology, University of North Texas, 1155 Union Circle no. 311280, TX 76203, USA

**Keywords:** adolescence, social reward, prosocial, antisocial, callous–unemotional traits, scale development

## Abstract

During adolescence, social interactions are a potent source of reward. However, no measure of social reward value exists for this age group. In this study, we adapted the adult Social Reward Questionnaire, which we had previously developed and validated, for use with adolescents. Participants aged 11–16 (*n* = 568; 50% male) completed the Social Reward Questionnaire—Adolescent Version (SRQ-A), alongside measures of personality traits—five-factor model (FFM) and callous–unemotional (CU) traits—for construct validity purposes. A confirmatory factor analysis of the SRQ-A supported a five-factor structure (Comparative Fit Index = 0.90; Root Mean Square Error of Approximation = 0.07), equating to five questionnaire subscales: enjoyment of Admiration, Negative Social Potency, Passivity, Prosocial Interactions and Sociability. Associations with FFM and CU traits were in line with what is seen for adult samples, providing support for the meaning of SRQ-A subscales in adolescents. In particular, adolescents with high levels of CU traits showed an ‘inverted’ pattern of social reward, in which being cruel is enjoyable and being kind is not. Gender invariance was also assessed and was partially supported. The SRQ-A is a valid, reliable measure of individual differences in social reward in adolescents.

## 1. Background

Adolescence describes the period of transition between childhood and adulthood when individuals undergo considerable psychological and physical change [1]. In particular, social cognition and behaviour change dramatically in this period, underpinned by the rapid development of the ‘social brain’, the network of brain areas involved in social information processing (e.g. [2]). Because of this, social relationships become increasingly salient in adolescence, particularly with regard to gaining approval and avoiding rejection from peers [3,4]. Additionally, during adolescence there is rapid development of the brain's dopaminergic system, which processes rewarding stimuli [5,6]. Together, the neural changes in social and reward processing networks mean that adolescents may find social interactions particularly motivating and influential, which can lead to more risky behaviour in the presence of peers (e.g. [7]). Understanding social reward processing in adolescents is therefore critical for understanding social behaviour and well-being in this age group.

Experimental evidence has documented the reward value of social stimuli in adolescence. For example, studies have found that adolescents’ performance on cognitive tasks are improved with positive social feedback such as smiling faces [8] or ‘thumbs up’ gestures [9], and such stimuli are subjectively rated as likeable [9]. Other research has indicated that socially rewarding stimuli may actually be more salient for adolescents than they are for adults. For example, distracting smiling faces impaired performance in a working memory task for adolescents (aged 12–14) but not adults, indicating that smiling faces may be especially salient for the adolescents [10].

Despite the theoretical and empirical data that indicate that social stimuli and interactions are an important source of reward for adolescents, to our knowledge, no research has attempted to empirically identify and categorize the different types of social interactions that adolescents find rewarding. Some researchers only discuss that social relationships, in general, become more rewarding in adolescence (e.g. [5]). Others evaluate a specific type of social reward, such as the presence of peers [11] or smiling faces [8,10]. However, as yet, there has not been a comprehensive assessment of the full range of social experiences that are rewarding for adolescents. There is also no existing measure, to our knowledge, that assesses individual differences in the reward value of social experiences for use in adolescents.

In adults, the social reward questionnaire (SRQ [12]) is a valid and reliable measure of individual differences in the value of different social rewards. This previous study used exploratory and confirmatory factor analyses (EFA and CFA) to identify six types of social reward: enjoyment of Admiration, Negative Social Potency, Passivity, Prosocial Interactions, Sexual Relationships and Sociability [12]. Each type of social reward equates to a subscale in the SRQ. These six subscales were not selected *a priori*, but were the result of the model that best fit the initial data set of 75 items. After the initial EFA supported this six-factor model, the best items in each factor were selected (those that loaded most strongly and unambiguously onto each factor) and the other items were discarded. This refined item set (23 items, 3–5 items for each of the 6 factors) were then subjected to a CFA. This CFA confirmed that a six-factor model fit the data well. The names of the subscales were chosen to reflect the content of the items within each factor (see [12] for more detail on the development of the adult SRQ).

The subscales in the adult SRQ cover the following domains of social reward: *Sexual Relationships,* the enjoyment of sexual intimacy; *Admiration*, the enjoyment of being flattered and gaining positive attention; *Negative Social Potency*, the enjoyment of being cruel, antagonistic and using others; *Passivity*, the enjoyment of giving others control and allowing them to make decisions; *Prosocial Interactions*, the enjoyment of having kind and reciprocal relationships; and *Sociability*, the enjoyment of engaging in group interactions. Each subscale has good psychometric properties and also showed a unique pattern of associations with external measures, providing support for the meaning of each subscale [12]. The primary aim of the present study is to modify the adult SRQ so that it can be used to assess individual differences in social reward value in adolescents.

The adult SRQ can be used as a tool to explore the experience of social reward in individuals who display problematic or unusual social behaviour [13,14]. For example, psychopathic traits—problematic personality traits including a lack of empathy and antisocial behaviour [15]—have been associated with an atypical pattern of social reward [13]. Specifically, adults with high levels of psychopathic traits display an ‘inverted’ pattern of social reward, in which they report that being cruel towards others is enjoyable and being kind is not. This is in stark contrast to the majority of typical individuals, who find cruelty aversive and experience affiliative interactions and relationships as a fundamental source of reward (e.g. [16]). These findings are potentially important when trying to understand the mechanisms behind the atypical social behaviour seen in psychopathy, i.e. the high levels of antisocial behaviour and low levels of affiliative, prosocial behaviour.

Psychopathic-type traits such as a lack of empathy and guilt can be detected in children and adolescents, and are termed callous–unemotional (CU) traits in this age group [17]. Young people with high levels of CU traits are at an elevated risk of having high levels of psychopathic traits when they become adults, and so CU traits are considered to be antecedents to adult psychopathy [18]. Like adults with psychopathic traits, adolescents with high levels of CU traits display problematic social behaviour. For example, compared with adolescents with low levels of CU traits, those with high levels of CU traits tend to endorse more antisocial solutions to achieve their goals, such as using aggression [19], and are more likely to bully others [20]. Unsurprisingly, their friendships tend to be shorter than those of typical adolescents [21]. It is important to understand possible mechanisms behind the problematic social behaviour seen in these adolescents, and one such mechanism is atypical social reward.

There is some limited existing evidence that adolescents with high levels of CU traits may have atypical processing of typically rewarding social stimuli such as happy faces [22,23]. In one study, adolescents with high levels of CU traits were less distracted by irrelevant happy faces compared with typically developing controls [23]. Other research has demonstrated that children with high levels of CU traits spend less time looking at their mothers’ faces, irrespective of the mothers’ behaviour [22]. Together, this research presents an interesting possibility that typically socially rewarding stimuli (such as happy faces) may have less reward value in adolescents with high levels of CU traits. However, social reward has not yet been systematically examined in relation to CU traits. It is important to understand the possible association between CU traits and social reward value in adolescents, as this may increase understanding of the mechanisms behind their callous and antisocial behaviour towards others.

### 1.1. The current study

Our primary aim in the current study was to validate the Social Reward Questionnaire—Adolescent Version (SRQ-A), an adapted version of the adult SRQ, in a sample of typical 11–16 year olds. We reasonably expect that the types of social interactions that are rewarding for adults (as shown in the adult SRQ) will also be important sources of social reward for adolescents. Specifically, receiving the approval of others (as measured by SRQ subscale *Admiration*) and engaging in meaningful reciprocal relationships (as measured by SRQ *Prosocial Interactions*) are likely to be particularly rewarding in adolescence, when peer approval and intimate friendships take on increasing importance [24,25]. Similarly, we expect the enjoyment of engaging in group interactions (as measured by SRQ *Sociability*) to be important at a time when individuals start spending more time with friends and reporting more positive affect from doing so [26]. We expect the enjoyment of being cruel, antagonistic and using others (as measured by SRQ *Negative Social Potency*) will also be relevant in adolescence, since some individuals in this age group report this reward as a motivation for bullying (e.g. [27]). We had no explicit expectation that the enjoyment of giving others control and allowing them to make decisions (as measured by SRQ *Passivity*) would be an important social reward in adolescence, but kept these items for the adolescent questionnaire as an exploratory hypothesis.

Two brief measures of personality traits were included for the purpose of construct validity: the Ten-Item Personality Inventory (TIPI [28]), a measure of five-factor model (FFM) personality traits, and the CU subscale of the Antisocial Process Screening Device (APSD [29]). We hypothesized that we would find similar associations in the current study to those found in our earlier studies with the adult SRQ [12,13]. For example, we hypothesized that the personality trait extraversion would be positively associated with enjoyment of admiration, and that openness to experience would be positively associated with enjoyment of sociability [12,13]. We also hypothesized that CU traits would show a pattern of ‘inverted’ social reward, in which adolescents with high levels of these traits report more enjoyment of negative social potency and less enjoyment of prosocial interactions, in line with our previous findings from adults with high levels of psychopathic traits [12,13].

## 2. Material and methods

### 2.1. Participants

Data were collected from two state secondary schools in Greater London: one in South London (*n* = 382) and one in East London (*n* = 196). Ten participants had more than 20% of the SRQ-A data missing, indicating that the questionnaire had not been answered carefully. These participants were removed from all further analyses, leaving a final sample of *n* = 568. Participants were 11–16 years old (mean = 12.89, s.d. = 1.18; *n* = 19 did not disclose age). The sample was 50.0% male (*n* = 284) and 47.4% female (*n* = 269); 2.7% (*n* = 15) of the sample did not disclose gender. Ethnicity data were not collected due to constraints imposed by the schools.

Both schools have a similar proportion of pupils claiming Free School Meal Entitlement, a useful proxy measure for pupil socioeconomic disadvantage (South London school: 17.10%; East London school: 17.70%; national average in England: 13.46%).

### 2.2. Permission to carry out fieldwork

Ethical approval covered data collection in schools.

### 2.3. Data collection

Participants completed the questionnaires by hand during their morning registration period. Participants completed questionnaires in their class group, but were sat separately to ensure their responses were private. Taking part took approximately 10 min and participants were not compensated. Data were entered into an SPSS (v. 20) database by two researchers; 10% of entries were cross-checked for accuracy.

### 2.4. Scale development

The items in the SRQ-A were taken from the adult SRQ, with some items removed or modified to ensure the content was appropriate for use with 11–16 year olds. These decisions were made based on discussions with a panel of six researchers with expertise in adolescent development. First, the *Sexual Relationships* subscale was removed in its entirety as its content is inappropriate for young adolescents. (This subscale consisted of three items: *I enjoy having erotic relationships; I enjoy having many sexual experiences; I enjoy having an active sex life.*) Therefore, the SRQ-A consisted of five subscales: *Admiration*, the enjoyment of being flattered and gaining positive attention; *Negative Social Potency*, the enjoyment of being cruel, antagonistic and using others; *Passivity*, the enjoyment of giving others control and allowing them to make decisions; *Prosocial Interactions*, the enjoyment of having kind and reciprocal relationships; and *Sociability*, the enjoyment of engaging in group interactions.

In addition, the wording of two items was simplified: ‘*I enjoy **achieving recognition** from others’* was changed to ‘*I enjoy **getting praise** from others’* and ‘*I enjoy feeling emotionally **connected** to someone’* was changed to ‘*I enjoy feeling emotionally **close** to someone’*. The final questionnaire has a Flesch–Kincaid Reading Grade Level of 6.64^1^ [30], indicating that the wording should be understood by pupils in Grade 6 (USA) and above, i.e. those aged 11 years and older.

### 2.5. Measures

In addition to the SRQ-A, participants completed the following questionnaires for the purposes of construct validity and to assess associations between the SRQ-A and CU traits. Brief measures were chosen due to constraints on testing time imposed by the schools.

#### 2.5.1. Ten-Item Personality Inventory

The TIPI [28] is a 10-item scale that measures the ‘Big Five’ personality traits (agreeableness, conscientiousness, extraversion, neuroticism and openness to experience; e.g. [31]). All items begin with ‘I see myself as’ and are followed by two descriptive items such as ‘anxious, easily upset’. Responses are given on a 1–7 scale (1, disagree strongly, 7 = agree strongly). The TIPI was originally validated in an adult sample, but has since been used with adolescents (e.g. [32,33]). We had several hypotheses: SRQ-A Prosocial Interactions would be positively associated with agreeableness and conscientiousness; SRQ-A Negative Social Potency would be negatively correlated with these traits; and SRQ-A Sociability would be positively correlated with extraversion.

#### 2.5.2. Callous–unemotional subscale of the Antisocial Process Screening Device

The CU [29] subscale is a 6-item measure, with each item scored from 0 to 2 (0 = not at all true, 1 = sometimes true, 2 = definitely true). This subscale measures CU traits, with items such as ‘You are concerned about the feelings of others’ and ‘You feel bad or guilty when you do something wrong’ (both reverse-coded). The self-report version of the APSD used here has good psychometric properties (e.g. [34]; although see [35]). Note that due to time constraints and the nature of our hypotheses, only the CU subscale was administered. We hypothesized that CU traits would be positively associated with SRQ-A Negative Social Potency and negatively associated with SRQ-A Prosocial Interactions, in line with findings with an adult measure of psychopathic traits [13].

### 2.6. Data analysis procedure

#### 2.6.1. Missing data strategy

Before any analyses were conducted, 10 participants were removed for having between 20 and 100% of SRQ data missing (mean = 41.50%, s.d. = 0.27%), as this indicated that the questionnaire had not been answered carefully. For all remaining analyses, all participants were retained, including those with less than 20% missing SRQ-A data. Participants with one or more items of missing questionnaire data (SRQ-A, TIPI or APSD; *n* = 106) did not differ from those without (*n* = 462) on gender ($\chi_{\left(1,n=553\right)}^{2}=1.36$, *p* = 0.24; *n* = 15 did not disclose gender) or age (*t*_547_ = −0.247, *p* = 0.80; *n* = 19 did not disclose age). Specific strategies for dealing with missing data are described in the following sections.

#### 2.6.2. Confirmatory factor analysis

To assess the latent structure of the social reward item set in adolescents, CFA was conducted on the 20-item SRQ-A using Mplus v. 7.1 [36]. The sample size (*n* = 568) was adequate for testing a model consisting of 50 parameters (i.e. 20 factor loadings, 20 error variances and 10 factor correlations). Specifically, the subjects-to-parameters ratio for the 20-item model is approximately 11 : 1, which is higher than the 10 : 1 minimum ratio recommended by Bentler and Chou [37].

We used the mean and variance-adjusted weighted least squares (WLSMV) estimation procedure as recommended for analysis of ordinal data [36]. Our intention was to assess whether the item set from adolescents showed the same factor structure (minus the Sexual Relationships factor) as that from adults [12]. The default in Mplus is to estimate latent models using all available data, including cases that have some missing values for some variables. Therefore, all available data were used for the CFA. The proportion of missing values for the current study was examined by a covariance coverage matrix, which provides an estimate of available observations for each pair of variables. The percentage of data present for each pair of variables ranged from 98 to 100%, indicating that the amount of missing data was minimal.

As recommended by Hu & Bentler [38], we used a two-index strategy to assess model fit: the incremental Comparative Fit Index (CFI) and the Root Mean Square Error of Approximation (RMSEA), an absolute fit index. We adopted the traditional CFI of 0.90 or above and RMSEA of 0.08 or below [39] as indicative of acceptable model fit. Our rationale is based on the fact that as model complexity increases, so does the difficulty of achieving conventional levels of model fit [40]. Thus, we chose to use conventional criteria to avoid falsely rejecting a viable latent variable model; we believed the use of conventional criteria was reasonable.

#### 2.6.3. Internal consistency

Cronbach α values and mean inter-item correlations (MICs) were measured to assess the internal consistency of each SRQ-A subscale. Given the limitations of Cronbach alpha and that it is not an indicator of scale unidimensionality [41], we relied more on the scale MICs to assess item homogeneity and internal consistency.

#### 2.6.4. Construct validity

Using SPSS (v. 20), Pearson correlational analyses were conducted to assess associations between SRQ-A subscales and measures of personality (TIPI and CU subscale of the APSD). Pairwise correlations were calculated to maximize the use of available data. Benjamini and Hochberg false discovery rate [42] was used to control for the probability of making a type I error on multiple comparisons.

#### 2.6.5. Test–retest reliability

In order to measure the stability of responses over time, a subset of participants from Sample 2 completed the SRQ twice, exactly one week apart. Pairwise Pearson correlational analyses were conducted to assess associations between subscale scores at the two time points, and Benjamini and Hochberg false discovery rate [42] was used to control for the probability of making a type I error on multiple comparisons.

#### 2.6.6. School clustering

Participants were from two different schools. We conducted a supplementary CFA that took into account the clustered nature of the data, in order to assess model fit while taking into account the degree of non-independence across cases.

#### 2.6.7. Testing measurement invariance across gender

We assessed two types of invariance: metric (in which item loadings are fixed but thresholds are free) and scalar (more stringent; in which both item loadings and thresholds are fixed). Before conducting formal multiple-group CFA, model fit for the five-factor SRQ-A model was first tested for the total sample, and then the subsamples of males and females were examined separately. Next, we conducted multi-group CFA (MG-CFA) to test for the two types of invariance across males and females.

To test for metric invariance, item loadings were constrained to be equal across the two genders, and we then assessed whether this model differed significantly from an unconstrained (i.e. configural) model in which both loadings and thresholds were free between the two groups. To test for scalar invariance, both item loadings and thresholds were constrained to be equal across gender, and this model was then compared with the configural model. (Note that items 1, 2 and 9 were omitted from the MG-CFA models because the female group did not have responses to all possible values (items 1 and 2: no females answered value 1; item 19: no females answered value 2 or 3).

If the incremental change in the CFI (ΔCFI) between the configural and the MG-CFA models is less than or equal to 0.01, this indicates that the two models within the comparison do not differ statistically in terms of fit [7]. This would suggest relatively good to strong measurement invariance across gender [43]. Since the SRQ-A items are ordinal and the WLSMV estimation procedure was used, we also used the Mplus DIFFTEST procedure to generate traditional *χ*^2^ difference tests.

## 3. Results

### 3.1. Confirmatory factor analysis

**Figure 1.**
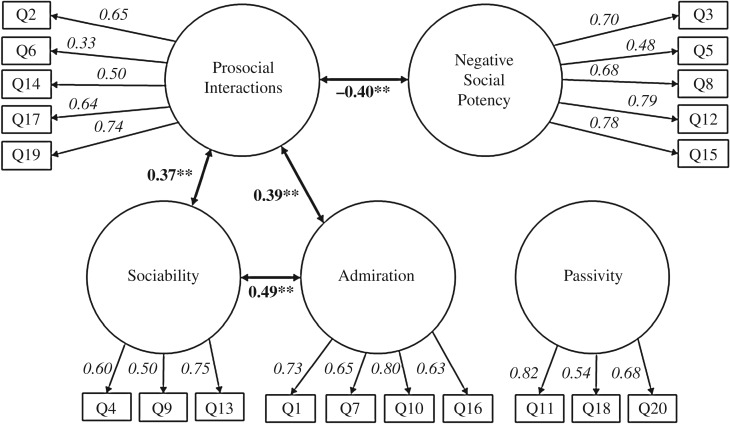
Social Reward Questionnaire—Adolescent Version (SRQ-A). Correlation coefficients are in bold, **p < 0.01, only significant correlations are shown; standardized factor loadings are in italics.

The FFM based on the adult version of the questionnaire achieved good fit using the data from the total sample of adolescents ($\chi_{\left(160\right)}^{2}=659.69,$ *p* < 0.001; CFI = 0.90; RMSEA = 0.07, 90% CI = 0.07–0.08). Factor loadings were in the range 0.33–0.82 (mean = 0.65, s.d. = 0.13) and are shown in table 1; a summary of the CFA results is shown in figure 1.

**Table 1.** Standardized factor loadings from the five-factor CFA.

factor	loading	item number
Admiration	0.73	1
	0.65	7
	0.80	10
	0.63	16
Negative Social Potency	0.70	3
	0.48	5
	0.68	8
	0.79	12
	0.78	15
Passivity	0.82	11
	0.54	18
	0.68	20
Prosocial Interactions	0.65	2
	0.33	6
	0.50	14
	0.64	17
	0.74	19
Sociability	0.60	4
	0.50	9
	0.75	13

### 3.2. Internal consistency

Cronbach α values and MICs were calculated for each subscale to assess internal consistency (table 2). Cronbach α values were in the range 0.56–0.74 (mean = 0.67, s.d. = 0.09). For some of the subscales, α falls below the cut-off point that is considered acceptable (0.70). However, Cronbach α is influenced by scale length, and these subscales contain only three to five items. It is also not a measure of scale unidimensionality. We therefore also calculated MICs, a measure of scale unidimensionality that is not affected by item number. MICs were in the range 0.25–0.43 (mean = 0.35, s.d. = 0.08). These fall within the acceptable range of 0.15–0.50 suggested by Clark & Watson [44]. All items showed good item-to-subscale-total correlations (range 0.60–0.82, mean = 0.71; s.d. = 0.07; table 2).

**Table 2.** Correlations, Cronbach α values, MICs and mean item-scale correlations (MISCs) for manifest factor totals (*n* = 568). Corrected *p*-values are shown and Cronbach α values appear in italics on the diagonal.

factor	1	2	3	4	5	MIC	MISC
1. Admiration	*0.74*					0.43	0.76
2. Negative Social Potency	0.05	*0.76*				0.39	0.72
3. Passivity	0.05	−0.07	*0.67*			0.40	0.77
4. Prosocial Interactions	0.39**	−0.40**	0.04	*0.60*		0.25	0.63
5. Sociability	0.49**	0.00	0.02	0.37**	*0.56*	0.30	0.73

**p < 0.01.

### 3.3. Test–retest reliability

A subset of participants completed the SRQ-A twice, 7 days apart (*n* = 46). To select participants to complete the SRQ-A twice, two classes were chosen at random from one of the schools. Data from five participants were excluded from the test–retest analysis: one participant answered ‘strongly disagree’ to 19/20 questions at Time 2, indicating that the questionnaire was not answered carefully; one participant had more than 20% of SRQ data missing at Time 1; and three participants who gave data at Time 1 were not available at Time 2. This left a final test–retest sample of *n* = 41, aged 11–13 (mean = 12.54, s.d. = 0.55). The sample was 36.60% (*n* = 15) male.

At each time point, subscale scores were calculated if participants had 50% or more valid data for that subscale (i.e. less than 50% missing data). Therefore, subscale scores were calculated for Admiration, Negative Social Potency and Prosocial Interactions if the participant had three or more valid answers (75% valid), and for Passivity and Sociability if the participants had two or more valid answers (66.66% valid). Pairwise Pearson correlations were conducted between SRQ-A subscale scores and Time 1 and Time 2. These were in the range 0.77–0.90 (mean = 0.81, s.d. = 0.06; all *p* < 0.001; table 3). These correlations indicate good test–retest reliability.

**Table 3.** Test–retest reliability: Pearson correlations between factor subtotal scores at Time 1 and Time 2 (time interval = 7 days).

factor	correlation between Time 1 and Time 2
Admiration	0.84
Negative Social Potency^a^	0.77
Passivity	0.78
Prosocial Interactions	0.90
Sociability	0.77

^a^*n* = 40; for all other correlations *n* = 41; all *p* < 0.001.

### 3.4. Construct validity: Ten-Item Personality Inventory and callous–unemotional subscale of Antisocial Process Screening Device

As described in the ‘Data analysis procedure’ section, subscale scores were calculated if participants had 50% or more valid data for that subscale (i.e. <50% missing data). Cronbach α values and MICs of the TIPI and CU subscale are reported in table 4. The Pearson correlational analyses were used to explore the pattern of associations between the five SRQ subscales, the TIPI personality subscales and the CU subscale of the APSD (see table 5; only corrected *p*-values are presented).

**Table 4.** Cronbach α values and MICs for CU scale and TIPI. Corrected *p*-values are shown and MICs for TIPI subscales consist of a single correlation (as each subscale is made up of two items only).

factor	Cronbach α	MIC
TIPI		
agreeableness	0.17	0.10
conscientiousness	0.23	0.13
extraversion	0.40	0.26
neuroticism	0.40	0.26
openness	0.29	0.17
CU traits	0.30	0.08

**Table 5.** Pearson correlations between SRQ subscales and external measures of personality and CU traits. All comparisons are corrected for multiple comparisons. Correlations of *p* < 0.05 after correcting for multiple comparisons are in italics.

		SRQ factor				
		admiration	negative social potency	passivity	prosocial interactions	sociability
personality trait						
agreeableness	*r*	0.06	−0.39**	0.07	0.28**	0.11*
	*n*	541	540	541	541	541
conscientiousness	*r*	0.19**	−0.20**	0.01	0.24**	0.07
	*n*	548	547	548	548	548
extraversion	*r*	0.24**	0.00	−0.17**	0.19**	0.29**
	*n*	549	548	549	549	549
neuroticism	*r*	0.07	−0.07	−0.02	0.01	0.05
	*n*	540	549	550	550	550
openness	*r*	0.20**	−0.15**	−0.01	0.30**	0.26**
	*n*	547	546	547	547	547
CU traits	*r*	−0.14**	0.39**	−0.12*	−0.37**	−0.08
	*n*	554	553	554	554	554

*p < 0.05, **p < 0.01.

Each SRQ-A subscale demonstrated a distinct pattern of associations with the personality subscales, indicating that each SRQ-A measures a relatively distinct aspect of social reward. Admiration was positively associated with conscientiousness, extraversion and openness; Negative Social Potency was negatively associated with agreeableness, conscientiousness and openness; Passivity was negatively associated with extraversion; Prosocial Interactions was positively associated with agreeableness, conscientiousness, extraversion and openness; and Sociability was positively associated with agreeableness, extraversion and openness. These associations were in line with hypotheses (see the ‘Measures’ section), and provide support for the meaning of each SRQ-A subscale.

As hypothesized, CU traits were positively associated with Negative Social Potency and negatively associated with Prosocial Interactions. In addition, CU traits were negatively associated with Admiration and Passivity. It is important to note that measures of internal consistency fell below accepted cut-offs for the TIPI and CU subscale, and associations between these measures and the SRQ-A should be made with this is mind. However, despite this, patterns of associations with these two measures are in line with those reported in adult SRQ data with more robust construct validity measure [12,13].

### 3.5. Descriptives

Means, standard deviations and minimum and maximum scores for each subscale are given in table 6.

**Table 6.** Descriptives for each subscale.

subscale	minimum	maximum	mean (s.d.)	*n*
Admiration	2.33	7.00	5.46 (1.06)	568
Negative Social Potency	1.00	7.00	2.77 (1.21)	567
Passivity	1.00	6.00	2.69 (1.16)	568
Prosocial Interactions	2.20	7.00	5.73 (0.79)	568
Sociability	1.00	7.00	5.40 (1.08)	568

### 3.6. School clustering

Given that the participants were clustered within one of two schools, we employed the COMPLEX analysis procedure available in Mplus for estimating model parameters when there is some degree of non-independence across the cases. These supplementary CFA results for the five-factor SRQ-A model were excellent (CFI = 0.98, RMSEA = 0.05), and thus provide further support for the model. Finally, to be comprehensive, we also ran a strict MG-CFA (equal loadings and thresholds), using the two schools as the grouping variable. The supplementary MG-CFA results were in-line with those conducted across gender (CFI = 0.92, RMSEA = 0.05) and provided strong evidence for parameter invariance across the two school settings.

### 3.7. Measurement invariance across gender

The MG-CFA results indicated that factor loadings could be constrained to be equal across gender without meaningful change in model fit (change in CFI > 0.01; table 7). This provides evidence for partial (metric) invariance, and indicates that the SRQ-A items discriminate social reward value similarly across male and female 11–16 year olds. (Although the χ^2^ difference test was significant, this test is sensitive to large sample sizes and is not recommended as the best means of assessing differences between two models [7,36]). Evidence for the more stringent scalar invariance was not found (i.e. change in CFI was at 0.01). However, the departure in model fit was by no means substantial, suggesting the degree of non-invariance in thresholds (i.e. endorsement likelihood) was relatively minor. Moreover, while the absence of strict scalar invariance indicates that the SRQ-A items are not always endorsed similarly across gender, when we examined the patterns of threshold differences by gender using plots, there did appear to be considerable similarity between females and males (see the electronic supplementary material including tables S1 and S2).

**Table 7.** Total sample and MG-CFA for the five-factor SRQ-A.

CFA model	CFI	RMSEA	*χ*^2^ diff	*p*-value
overall model fit by total, female and male samples				
total^a^	0.93	0.06	—	—
female	0.93	0.06	—	—
male	0.90	0.06	—	—
multi-group analyses: tests for invariance across males and females				
configural (free loadings and thresholds)	0.91	0.06	—	—
metric (fixed loadings, free thresholds)	0.91	0.06	$\chi_{\left(11\right)}^{2}=43.52$	0.00
scalar (fixed loadings and thresholds)	0.90	0.06	$\chi_{\left(90\right)}^{2}=147.23$	0.00

^a^The model-fit statistics reported here differ slightly from those reported in the main CFA because items 1, 2 and 9 were omitted from these MG-CFA models (due to the limited variance for these items in the female sample).

## 4. Discussion

This paper describes the development of the 20-item SRQ-A. The SRQ-A is a valid and reliable measure of individual differences in the value of social rewards, for use with 11–16 year olds. CFA demonstrated that an FFM based on the adult SRQ [12] fitted the adolescent data well, and measurement invariance analyses indicated that this model applied to both males and females. The five latent factors are represented by five manifest variable subscales of social reward domains: Admiration, Negative Social Potency, Passivity, Prosocial Interactions and Sociability. Further analyses demonstrated that the SRQ-A has good internal consistency, test–retest reliability and construct validity. In particular, we found that CU traits were positively associated with the Negative Social Potency subscale (enjoyment of being cruel and antagonistic) and negatively associated with the Prosocial Interactions subscale (enjoyment of being kind and prosocial). This indicates that adolescents with high levels of CU traits show a pattern of ‘inverted’ social reward, in which being cruel is enjoyable and being kind is not, much like adults with high levels of psychopathic traits [13].

Associations between the SRQ-A subscales and personality domains provide support for the meaning of each subscale and indicate that they are capturing distinct types of socially rewarding interactions. For example, the personality trait extraversion was positively associated with SRQ-A Admiration. Individuals with high levels of extraversion are sociable, friendly and seek out social interactions (e.g. [45]), so the positive association with the enjoyment of admiration found here provides support that the Admiration subscale is capturing enjoyment of positive social attention. Agreeableness, a personality trait describing warmth and kindness (e.g. [45]), was positively associated with SRQ-A Prosocial Interactions and negatively associated with SRQ-A Negative Social Potency. Agreeableness has previously been associated with motivation to use prosocial tactics, such as compromise, to resolve conflict [46]. Its positive association with SRQ-A Prosocial Interactions provides support that this subscale is capturing enjoyment of behaving prosocially, and its negative association with SRQ-A Negative Social Potency provides support that this subscale captures the reward value of behaving in an antagonistic and antisocial manner towards others.

Analyses with CU traits indicate that adolescents with high levels of CU traits show a pattern of ‘inverted’ social reward, in a similar manner to adults with high levels of psychopathic traits [13]. Specifically, adolescents with high levels of CU traits report more enjoyment from being cruel, callous and antagonistic towards others, and less enjoyment from having affiliative, prosocial exchanges with others. This evidence of increased enjoyment from antisocial behaviour is in line with other research showing a moderate association between psychopathic and sadistic personality traits in adolescents [47]—indicating that adolescents with high levels of psychopathic-type traits find some enjoyment in hurting others. The positive association reported here between CU traits and SRQ-A Negative Social Potency suggests that the increased levels of antisocial behaviour seen in adolescents with high levels of CU traits (e.g. [48]) may be motivated partly by the reward value that this behaviour has for these individuals.

Equally, the negative association between CU traits and enjoyment of prosocial relationships may provide an important explanation for why these individuals are less likely to affiliate with and behave prosocially towards others [17,22] and have long-term friendships [21]. Although low levels of prosocial behaviour and affiliation are well documented in descriptions of those with high levels of CU traits, the findings presented here are some of the first to examine *why* this behaviour is reduced in these individuals. The current findings present an interesting possibility that prosocial interactions and relationships may simply not be as enjoyable—or enjoyable at all—for adolescents with high levels of CU traits. If this is the case, it makes intuitive sense that these individuals are not motivated to engage in these behaviours (unless, of course, there is some consequence that *is* rewarding for them).

This ‘inverted’ pattern of social reward—where being cruel is enjoyable and being kind is not—should be taken into account when considering interventions to reduce levels of CU traits. For example, it would be interesting to explore whether it is possible to modify the low levels of social reward experienced from prosocial interactions by these adolescents. Some authors have suggested that a particular emphasis on parental warmth and responsiveness may be important when designing interventions for adolescents with high levels of CU traits (e.g. [49,50]), and in line with this, children both with and without high levels of CU traits responded equally well to an intervention that focused on increasing parental positive reinforcement to encourage prosocial behaviour [51]. More longitudinal, randomized control studies are needed to explore if such parental interventions are effective at reducing CU traits, and importantly if the mechanism of change is associated with a change in the reward value of prosocial exchanges. Equally, it would be important to understand whether the increased reward value of antisocial behaviour could be reduced, for example, by emphasizing potential costs to the individual of behaving antisocially, or potential gains of behaving prosocially.

It is important to note differences between the current adolescent sample and the previous adult sample [13] with regard to associations between the SRQ subscales and psychopathic/CU traits. For example, the current study showed a modest *negative* association between CU traits and both enjoyment of admiration and enjoyment of passivity. In contrast, the previous adult sample showed *positive* associations between interpersonal psychopathic traits and enjoyment of admiration, and between interpersonal, lifestyle and antisocial psychopathic traits and enjoyment of passivity [13]. This presents an interesting possibility that gaining approval and praise (Admiration) and allowing others to take the lead (Passivity) may have different reward values for adults with high levels of psychopathic traits compared with adolescents with high levels of CU traits. For example, for adults, the Admiration subscale may be interpreted as social attention that is flattering and useful for manipulating others. Adolescents, on the other hand, may interpret the items in the Admiration subscale as indicative of gaining approval from authority figures such as teachers and parents, which may be undesirable to them. Similarly, passivity may be enjoyable for adults with high levels of psychopathic traits as allowing others to make decisions means less effort for the individual and more opportunity to be a ‘free loader’ [13]. By contrast, for adolescents, passivity may be interpreted as submission to authority. This may be particularly undesirable for adolescents with high levels of CU traits, who tend to rebel [17]. We acknowledge the speculative nature of these interpretations; further investigations are necessary to better understand how adults and adolescents interpret the meaning of the different SRQ subscales.

Additionally, it is interesting to interpret the negative association between CU traits and the Admiration subscale in the context of what is known to be rewarding for typical adolescents. The current findings suggest that, as CU traits increase, admiration or approval from others is less rewarding. This is particularly interesting as this source of reward is considered to be so potent for typical adolescents [3,4]*.* As the SRQ-A asks participants to answer the questions in relation to *all* people in their lives, it is not clear whether this reduced reward value of admiration relates to interactions with peers, parents, teachers or some combination of these. It would be interesting to explore this further in future studies to better understand in what way gaining admiration and approval from others, a salient reward for most adolescents, may be less rewarding for those with high levels of CU traits.

### 4.1. Limitations

There are several limitations of the current study. First, the items were taken from the adult SRQ [12], after the Sexual Relationships subscale was dropped and the wording of two items was adjusted. For the adult SRQ, the final items were chosen after an EFA was conducted on a wider item set (75 items); the items in the final questionnaire were those that loaded most strongly and unambiguously onto their respective factors. In the adolescent version described here, these final adult items (with one subscale removed and two items slightly adjusted) were used to conduct a CFA with an adolescent sample. It is important to consider that if an entirely data-driven approach was used with adolescents, in which an EFA was first conducted on a wider item set, different factors and items may have been discovered. However, the FFM taken from the adult SRQ did have good model fit with the adolescent sample. This indicates that, although an entirely data-driven approach was not used, the FFM used here still captures meaningful aspects of social reward in adolescents; this is also indicated by associations between the SRQ-A subscales and external variables.

Evidence for partial (metric) invariance was found across males and females, but not for strict scalar invariance. This indicates that while the FFM captures social reward value for both males and females, and the items discriminate equally well between males and females, the threshold at which an item is endorsed may differ between genders. Nevertheless, invariance across groups is often more a matter of a degree as opposed to all-or-nothing [52]. In this sense, the pattern of item endorsements between females and males evidenced a fair degree of concordance. Further investigations with the SRQ-A using item-level analyses would be beneficial to better understand the nuances of how these items capture social reward value for each gender.

It is important to note that the internal consistency of the two external measures in this paper was low. Future studies should assess associations between the SRQ-A and other measures of personality traits with better internal consistency. Assessing associations between the SRQ-A and a more detailed measure of CU traits, such as the Inventory of Callous–Unemotional Traits (ICU [53]), would be particularly informative. The ICU is a multi-dimensional measure that assesses three facets of CU traits: callous, uncaring and unemotional features. Owing to time limits imposed by the schools in the current study, a brief measure of CU traits was used. Analysis using the ICU, which typically has higher reports of internal consistency [53], would allow the relationship between social reward value and CU traits to be explored in more detail. It is also important to note that the test–retest sample was from only one school. The psychometric properties of the SRQ-A will be strengthened by further assessments of its test–retest reliability in a wider sample of participants.

A final limitation is the absence of data on ethnicity and socioeconomic status (SES) for this sample. The lack of this information may limit the generalizability of the current findings. It is critical that future investigations collect more detailed information about ethnicity and SES in order to more comprehensively assess the construct of social reward in adolescence.

## 5. Conclusion

In this study, we describe the development and validation of the 20-item SRQ-A. The study provides initial data to support the validity and reliability of a measure to assess individual differences in the value of social rewards, for use with 11–16 year olds. Using CFA, we demonstrated that an FFM, based on the adult version of the SRQ, had good model fit with the adolescent sample. These five factors equate to the five subscales of the questionnaire: Admiration, Negative Social Potency, Passivity, Prosocial Interactions and Sociability. The questionnaire assesses individual differences in the reward value experienced from each of these social interaction domains. In addition, we presented analyses that provided initial support for the construct validity, internal reliability, test–retest reliability and partial gender invariance of the SRQ-A. In sum, the SRQ-A is a valid and reliable measure of individual differences in the value of social reward, for use in adolescent populations.

## Supplementary Material

Information about thresholds Uploaded as ‘Supplementary material - thresholds.doc’; details breakdown of item thresholds by gender (as part of gender invariance analysis)

## Supplementary Material

Adolescent SRQ dataset

## Footnotes

1 The Flesch–Kincaid Grade Level is computed using the following calculation: Grade Level = 0.39(Total words/Total sentences) + 11.8(Total syllables/Total words) − 15.59.

## Data accessibility

The dataset supporting this article is available as the electronic supplementary material. The dataset is also freely available for public use at Dryad: http://dx.doi.org/10.5061/dryad.n399g [54].

## References

[1] Blakemore S-J, Choudhury S 2006 Development of the adolescent brain: implications for executive function and social cognition. J. Child Psychol. Psychiatry 47, 296–312. (doi:10.1111/j.1469-7610.2006.01611.x) 16492261 10.1111/j.1469-7610.2006.01611.x

[2] Blakemore S-J 2008 The social brain in adolescence. Nat. Rev. Neurosci. 9, 267–277. (doi:10.1038/nrn2353) 18354399 10.1038/nrn2353

[3] Jones RM et al. 2014 Adolescent-specific patterns of behavior and neural activity during social reinforcement learning. Cogn. Affect. Behav. Neurosci. 14, 683–697. (doi:10.3758/s13415-014-0257-z) 24550063 10.3758/s13415-014-0257-z PMC4127887

[4] Sebastian C, Viding E, Williams KD, Blakemore S-J 2010 Social brain development and the affective consequences of ostracism in adolescence. Brain Cogn. 72, 134–145. (doi:10.1016/j.bandc.2009.06.008) 19628323 10.1016/j.bandc.2009.06.008

[5] Davey CG, Yücel M, Allen NB 2008 The emergence of depression in adolescence: development of the prefrontal cortex and the representation of reward. Neurosci. Biobehav. Rev. 32, 1–19. (doi:10.1016/j.neubiorev.2007.04.016) 17570526 10.1016/j.neubiorev.2007.04.016

[6] Steinberg L 2010 A dual systems model of adolescent risk-taking. Dev. Psychobiol. 52, 216–224. (doi:10.1002/dev.20445) 20213754 10.1002/dev.20445

[7] Cheung GW, Rensvold RB 2002 Evaluating goodness-of-fit indexes for testing measurement invariance. Struct. Equ. Model. 9, 233–255. (doi:10.1207/S15328007SEM0902_5)

[8] Kohls G, Peltzer J, Herpertz-Dahlmann B, Konrad K 2009 Differential effects of social and non-social reward on response inhibition in children and adolescents. Dev. Sci. 12, 614–625. (doi:10.1111/j.1467-7687.2009.00816.x) 19635087 10.1111/j.1467-7687.2009.00816.x

[9] Demurie E, Roeyers H, Baeyens D, Sonuga-Barke E 2012 The effects of monetary and social rewards on task performance in children and adolescents: liking is not enough. Int. J. Methods Psychiatr. Res. 21, 301–310. (doi:10.1002/mpr.1370) 23148022 10.1002/mpr.1370 PMC6878378

[10] Cromheeke S, Mueller SC 2015 The power of a smile: stronger working memory effects for happy faces in adolescents compared to adults. Cogn. Emot. 30, 288–301. (doi:10.1080/02699931.2014.997196) 25650124 10.1080/02699931.2014.997196

[11] Chein J, Albert D, O'Brien L, Uckert K, Steinberg L 2011 Peers increase adolescent risk taking by enhancing activity in the brain's reward circuitry. Dev. Sci. 14, 1–10. (doi:10.1111/j.1467-7687.2010.01035.x) 21499511 10.1111/j.1467-7687.2010.01035.x PMC3075496

[12] Foulkes L, Viding E, McCrory E, Neumann CS 2014 Social Reward Questionnaire (SRQ): development and validation. Front. Psychol. 11, 205 (doi:10.3389/fpsyg.2014.00201) 10.3389/fpsyg.2014.00201 PMC3949132 24653711

[13] Foulkes L, McCrory EJ, Neumann CS, Viding E 2014 Inverted social reward: associations between psychopathic traits and self-report and experimental measures of social reward. PLoS ONE 9, e0106000 (doi:10.1371/journal.pone.0106000) 10.1371/journal.pone.0106000 PMC4146585 25162519

[14] Foulkes L, Bird G, Gökçen E, McCrory E, Viding E 2015 Common and distinct impacts of autistic traits and alexithymia on social reward. PLoS ONE 10, e0121018 (doi:10.1371/journal.pone.0121018) 25853670 10.1371/journal.pone.0121018 PMC4390314

[15] Hare RD 2003 Manual for the revised psychopathy checklist, 2nd edn Toronto, ON: Multi-Health Systems.

[16] Esch T 2005 The neurobiology of love. Neuroendocrinol. Lett. 26, 175–192. 15990719

[17] Frick PJ, Ray JV, Thornton LC, Kahn RE 2013 Can callous-unemotional traits enhance the understanding, diagnosis and treatment of serious conduct problems in children and adolescents? A comprehensive review. Psychol. Bull. 140, 1–57. (doi:10.1037/a0033076) 23796269 10.1037/a0033076

[18] Lynam DR, Caspi A, Moffitt TE, Loeber R, Stouthamer-Loeber M 2007 Longitudinal evidence that psychopathy scores in early adolescence predict adult psychopathy. J. Abnorm. Psychol. 116, 155–165. (doi:10.1037/0021-843X.116.1.155) 17324026 10.1037/0021-843X.116.1.155 PMC3335348

[19] Pardini D 2011 Perceptions of social conflicts among incarcerated adolescents with callous-unemotional traits: ‘You're going to pay. It's going to hurt, but I don't care’. J. Child Psychol. Psychiatry 52, 248–255. (doi:10.1111/j.1469-7610.2010.02336.x) 21073459 10.1111/j.1469-7610.2010.02336.x PMC3034798

[20] Viding E, Simmonds E, Petrides KV, Frederickson N 2009 The contribution of callous-unemotional traits and conduct problems to bullying in early adolescence. J. Child Psychol. Psychiatry 50, 471–481. (doi:10.1111/j.1469-7610.2008.02012.x) 19207635 10.1111/j.1469-7610.2008.02012.x

[21] Muñoz LC, Kerr M, Besic N 2008 The peer relationships of youths with psychopathic personality traits a matter of perspective. Crim. Justice Behav. 35, 212–227. (doi:10.1177/0093854807310159)

[22] Dadds MR, Allen JL, McGregor K, Woolgar M, Viding E, Scott S 2014 Callous-unemotional traits in children and mechanisms of impaired eye contact during expressions of love: a treatment target? J. Child Psychol. Psychiatry 55, 771–780. (doi:10.1111/jcpp.12155) 24117894 10.1111/jcpp.12155

[23] Hodsoll S, Lavie N, Viding E 2014 Emotional attentional capture in children with conduct problems: the role of callous-unemotional traits. Front. Hum. Neurosci. 8, 570 (doi:10.3389/fnhum.2014.00570) 25206326 10.3389/fnhum.2014.00570 PMC4143881

[24] Buhrmester D 1990 Intimacy of friendship, interpersonal competence, and adjustment during preadolescence and adolescence. Child Dev. 61, 1101–1111. (doi:10.2307/1130878) 2209180

[25] Westenberg P, Drewes MJ, Goedhart AW, Siebelink BM, Treffers PD 2004 A developmental analysis of self-reported fears in late childhood through mid-adolescence: social-evaluative fears on the rise? J. Child Psychol. Psychiatry 45, 481–495. (doi:10.1111/j.1469-7610.2004.00239.x) 15055368 10.1111/j.1469-7610.2004.00239.x

[26] Larson R, Richards MH 1991 Daily companionship in late childhood and early adolescence: changing developmental contexts. Child Dev. 62, 284–300. (doi:10.2307/1131003) 2055123 10.1111/j.1467-8624.1991.tb01531.x

[27] Bosacki SL, Marini ZA, Dane AV 2006 Voices from the classroom: pictorial and narrative representations of children's bullying experiences. J. Moral Educ. 35, 231–245. (doi:10.1080/03057240600681769)

[28] Gosling SD, Rentfrow PJ, Swann WB 2003 A very brief measure of the Big-Five personality domains. J. Res. Pers. 37, 504–528. (doi:10.1016/S0092-6566(03)00046-1)

[29] Frick PJ, Hare RD 2001 The antisocial process screening device (APSD). Toronto, ON: Multi-Health Systems.

[30] Kincaid JP, Fishburne RP Jr, Rogers RL, Chissom BS 1975 Derivation of new readability formulas (Automated Readability Index, Fog Count and Flesch Reading Ease Formula) for navy enlisted personnel. Research Branch report 8-75. Memphis, TN: Naval Air Station.

[31] Costa PT, McCrae RR 1992 Four ways five factors are basic. Pers. Individ. Dif. 13, 653–665. (doi:10.1016/0191-8869(92)90236-I)

[32] Erol RY, Orth U 2011 Self-esteem development from age 14 to 30 years: a longitudinal study. J. Pers. Soc. Psychol. 101, 607–619. (doi:10.1037/a0024299) 21728448 10.1037/a0024299

[33] Harden KP, Tucker-Drob EM 2011 Individual differences in the development of sensation seeking and impulsivity during adolescence: further evidence for a dual systems model. Dev. Psychol. 47, 739–746. (doi:10.1037/a0023279) 21534657 10.1037/a0023279

[34] Munoz LC, Frick PJ 2007 The reliability, stability, and predictive utility of the self-report version of the Antisocial Process Screening Device. Scand. J. Psychol. 48, 299–312. (doi:10.1111/j.1467-9450.2007.00560.x) 17669220 10.1111/j.1467-9450.2007.00560.x

[35] Poythress NG, Douglas KS, Falkenbach D, Cruise K, Lee Z, Murrie DC, Vitacco M 2006 Internal consistency reliability of the self-report Antisocial Process Screening Device. Assessment 13, 107–113. (doi:10.1177/1073191105284279) 16443722 10.1177/1073191105284279

[36] Muthén LK, Muthén BO 2012 Mplus. The comprehensive modelling program for applied researchers: user's guide. Los Angeles, CA: Muthén and Muthén.

[37] Bentler PM, Chou C-P 1987 Practical issues in structural modeling. Sociol. Methods Res. 16, 78–117. (doi:10.1177/0049124187016001004)

[38] Hu L, Bentler PM 1999 Cutoff criteria for fit indexes in covariance structure analysis: conventional criteria versus new alternatives. Struct. Equation Modeling 6, 1–55. (doi:10.1080/10705519909540118)

[39] West SG, Taylor AB, Wu W 2012 Model fit and model selection in structural equation modeling. In Handbook of structural equation modeling (ed. Hoyle RH), pp. 209–231. New York, NY: Guildford Press.

[40] Marsh HW, Hau K-T, Wen Z 2004 In search of golden rules: comment on hypothesis-testing approaches to setting cutoff values for fit indexes and dangers in overgeneralizing Hu and Bentler's (1999) findings. Struct. Equation Modeling 11, 320–341. (doi:10.1207/s15328007sem1103_2)

[41] Schmitt N 1996 Uses and abuses of coefficient alpha. Psychol. Assessment 8, 350–353. (doi:10.1037/1040-3590.8.4.350)

[42] Benjamini Y, Hochberg Y 1995 Controlling the false discovery rate: a practical and powerful approach to multiple testing. J. R. Stat. Soc. Series B (Methodol) 1, 289–300.

[43] Mokros A, Neumann CS, Stadtland C, Osterheide M, Nedopil N, Hare RD 2011 Assessing measurement invariance of PCL-R assessments from file reviews of North American and German offenders. Int. J. Law. Psychiatry 34, 56–63. (doi:10.1016/j.ijlp.2010.11.009) 21122915 10.1016/j.ijlp.2010.11.009

[44] Clark LA, Watson D 1995 Constructing validity: basic issues in objective scale development. Psychol. Assessment 7, 309–319. (doi:10.1037/1040-3590.7.3.309) 10.1037/pas0000626 PMC6754793 30896212

[45] McCrae RR, Costa PT 1987 Validation of the five-factor model of personality across instruments and observers. J. Pers. Soc. Psychol. 52, 81 (doi:10.1037/0022-3514.52.1.81) 3820081 10.1037//0022-3514.52.1.81

[46] Jensen-Campbell LA, Graziano WG 2001 Agreeableness as a moderator of interpersonal conflict. J. Pers. 69, 323–362. (doi:10.1111/1467-6494.00148) 11339802 10.1111/1467-6494.00148

[47] Chabrol H, Van Leeuwen N, Rodgers R, Séjourné N 2009 Contributions of psychopathic, narcissistic, Machiavellian, and sadistic personality traits to juvenile delinquency. Pers. Individ. Dif. 47, 734–739. (doi:10.1016/j.paid.2009.06.020)

[48] Rowe R, Maughan B, Moran P, Ford T, Briskman J, Goodman R 2010 The role of callous and unemotional traits in the diagnosis of conduct disorder. J. Child Psychol. Psychiatry 51, 688–695. (doi:10.1111/j.1469-7610.2009.02199.x) 20039995 10.1111/j.1469-7610.2009.02199.x

[49] Frick PJ, White SF 2008 Research review: the importance of callous-unemotional traits for developmental models of aggressive and antisocial behavior. J. Child Psychol. Psychiatry 49, 359–375. (doi:10.1111/j.1469-7610.2007.01862.x) 18221345 10.1111/j.1469-7610.2007.01862.x

[50] Hyde LW et al. 2016 Heritable and nonheritable pathways to early callous-unemotional behaviors. Am. J. Psychiatry 173, 903–910. (doi:10.1176/appi.ajp.2016.15111381) 27056607 10.1176/appi.ajp.2016.15111381 PMC5008992

[51] Hawes DJ, Dadds MR 2005 The treatment of conduct problems in children with callous-unemotional traits. J. Consult. Clin. Psychol. 73, 737 (doi:10.1037/0022-006X.73.4.737) 16173862 10.1037/0022-006X.73.4.737

[52] Neumann CS, Schmitt DS, Carter R, Embley I, Hare RD 2012 Psychopathic traits in females and males across the globe. Behav. Sci. Law 30, 557–574. (doi:10.1002/bsl.2038) 22996170 10.1002/bsl.2038

[53] Essau CA, Sasagawa S, Frick PJ 2006 Callous-unemotional traits in a community sample of adolescents. Assessment 13, 454–469. (doi:10.1177/1073191106287354) 17050915 10.1177/1073191106287354

[54] Foulkes L, Neumann CS, Roberts R, McCrory E, Viding E 2017 Data from: Social Reward Questionnaire—Adolescent Version and its association with callous–unemotional traits. Dryad Digital Repository. (http://dx.doi.org/10.5061/dryad.n399g) 10.1098/rsos.160991 PMC5414254 28484617

