# Supplementary material for: Social Reward Questionnaire—Adolescent Version and its association with callous–unemotional traits
Source: R Soc Open Sci. 2017 Apr 19;4(4):160991. doi: 10.1098/rsos.160991 (PMC5414254; doi:10.1098/rsos.160991)
Supplement: Information about thresholds Uploaded as ‘Supplementary material - thresholds.doc’; details breakdown of item thresholds by gender (as part of gender invariance analysis) [file rsos160991supp1.docx]

**Thresholds**

A threshold is the predicted value of the underlying trait or factor that a respondent requires in order to transfer from one response value on the categorical outcome variable to the next (e.g. from 1 to 2; Muthen & Muthen, 2010). Higher thresholds indicate that a greater amount of a latent trait is needed before a particular item response is given (Reise, 1999).

For the SRQ-A, there are seven response options (1-7 Likert scale), and therefore six thresholds. In Figures 1-6, each threshold for each item is plotted, for males and females separately. (Please note that items 1, 2 and 19 were excluded from analysis as females did not use all response options for these three items.)

**Figure 1.** Threshold 1: Response 2 ('Disagree') over 1 ('Strongly Disagree')

**Figure 2.** Threshold 2: Response 3 ('Slightly disagree') over 2 ('Disagree')

**Figure 3.** Threshold 3: Response 4 ('Neutral') over 3 ('Slightly disagree')

**Figure 4.** Threshold 4: Response 5 ('Slightly agree') over 4 ('Neutral')

**Figure 5.** Threshold 5: Response 6 ('Agree') over 5 ('Slightly agree')

**Figure 6.** Threshold 6: Response 7 ('Strongly agree) over 6 ('Agree')

Table S1

*Pearson correlations between SRQ subscales and external measures of personality and CU traits for boys only*

|  |  | SRQ factor | | | | |
| --- | --- | --- | --- | --- | --- | --- |
|  |  | Admiration | Negative Social Potency | Passivity | Prosocial Interactions | Sociability |
| *Personality trait* |  |  |  |  |  |  |
| *Agreeableness* | r | -.03 | **-.32**** | -.01 | **.24**** | .02 |
|  | N | 269 | 269 | 269 | 269 | 269 |
|  |  |  |  |  |  |  |
| *Conscientiousness* | r | **.18**** | -.13 | .00 | **.23**** | .05 |
|  | N | 272 | 272 | 272 | 272 | 272 |
|  |  |  |  |  |  |  |
| *Extraversion* | r | **.26**** | -.05 | -.09 | **.25**** | **.34**** |
|  | N | 272 | 272 | 272 | 272 | 272 |
|  |  |  |  |  |  |  |
| *Neuroticism* | r | .02 | -.10 | -.14 | .00 | -.01 |
|  | N | 272 | 272 | 272 | 272 | 272 |
|  |  |  |  |  |  |  |
| *Openness* | r | **.22**** | **-.19**** | .03 | **.33**** | **.31**** |
|  | N | 272 | 272 | 272 | 272 | 272 |
|  |  |  |  |  |  |  |
| *CU traits* | r | **-.19**** | **.31**** | -.09 | **-.38**** | -.03 |
|  | N | 272 | 272 | 272 | 272 | 272 |

*Note*. All comparisons corrected for multiple comparisons. Correlations of p<.05 after correcting for multiple comparisons are in bold; *p<.05, **p<.01

Table S2

*Pearson correlations between SRQ subscales and external measures of personality and CU traits for girls only*

|  |  | SRQ factor | | | | |
| --- | --- | --- | --- | --- | --- | --- |
|  |  | Admiration | Negative Social Potency | Passivity | Prosocial Interactions | Sociability |
| *Personality trait* |  |  |  |  |  |  |
| *Agreeableness* | r | **.16*** | **-.45**** | **.19**** | **.30**** | **.22**** |
|  | N | 259 | 258 | 259 | 259 | 259 |
|  |  |  |  |  |  |  |
| *Conscientiousness* | r | **.20**** | **-.26**** | .04 | **.26**** | .10 |
|  | N | 262 | 261 | 262 | 262 | 262 |
|  |  |  |  |  |  |  |
| *Extraversion* | r | **.25**** | .09 | **-.22**** | **.15*** | **.26**** |
|  | N | 264 | 263 | 264 | 264 | 264 |
|  |  |  |  |  |  |  |
| *Neuroticism* | r | **.18**** | -.11 | .04 | **.15*** | .10 |
|  | N | 265 | 264 | 265 | 265 | 265 |
|  |  |  |  |  |  |  |
| *Openness* | r | **.21**** | -.09 | -.02 | **.31**** | **.20**** |
|  | N | 262 | 261 | 262 | 262 | 262 |
|  |  |  |  |  |  |  |
| *CU traits* | r | -.07 | **.45**** | **-.20**** | **-.33**** | **-.16*** |
|  | N | 267 | 266 | 267 | 267 | 267 |

*Note*. All comparisons corrected for multiple comparisons. Correlations of p<.05 after correcting for multiple comparisons are in bold; *p<.05, **p<.01

**References**

Reise, S. P. (1999). Personality measurement issues viewed through the eyes of IRT. In Embretson, S. E., and Hershberger, S. L. (Eds.), *The New Rules of Measurement: What Every Psychologist and Educator Should Know* (pp 219-242), Mahwah, NJ: Lawrence Erlbaum.
